# Systemic Inflammatory Index Is a Novel Predictor of Intubation Requirement and Mortality after SARS-CoV-2 Infection

**DOI:** 10.3390/pathogens10010058

**Published:** 2021-01-11

**Authors:** Sajjad Muhammad, Igor Fischer, Soheil Naderi, Morteza Faghih Jouibari, Sheikhrezaei Abdolreza, Ehsan Karimialavijeh, Sara Aslzadeh, Mahsa Mashayekhi, Mohaddeseh Zojaji, Ulf Dietrich Kahlert, Daniel Hänggi

**Affiliations:** 1 Department of Neurosurgery, Medical Faculty, Heinrich-Heine-University, Moorenstrasse 5, 40225 Düsseldorf, Germany; igor.fischer@med.uni-duesseldorf.de (I.F.); Ulf.Kahlert@med.uni-duesseldorf.de (U.D.K.); Daniel.Haenggi@med.uni-duesseldorf.de (D.H.); 2 Department of Neurosurgery, Tehran University of Medical Sciences, Theran 1417613151, Iran; snaderi@sina.tums.ac.ir (S.N.); Dr_sheikhrezaei@yahoo.com (S.A.); 3 Neurosurgery Department, Shariati Hospital, Tehran University of Medical Sciences, Theran 1417613151, Iran; m-faghihj@sina.tums.ac.ir; 4 Department of Emergency Medicine, Tehran University of Medical Sciences, Theran 1417613151, Iran; drkarimi86@gmail.com; 5 Department of Neurology, Sina Hospital, Tehran University of Medical Sciences, Tehran 1193653471, Iran; Saraaslzadeh@gmail.com; 6 Department of Internal Medicine, Tabriz University of Medical Sciences, Tabriz 5166/15731, Iran; mahsamashayekhi69@gmail.com; 7 Department of Internal Medicine, Qom University of Medical Sciences, Qom 371364967, Iran; drzojaji@gmail.com

**Keywords:** coronavirus 2019 disease, personalized medicine, infections disease, health care management, economics, neurosurgery, practical guidelines, pandemic

## Abstract

Coronavirus disease 2019 (COVID-19), with an increasing number of deaths worldwide, has created a tragic global health and economic emergency. The disease, caused by severe acute respiratory syndrome coronavirus 2019 (SARS-CoV-19), is a multi-system inflammatory disease with many of COVID-19-positive patients requiring intensive medical care due to multi-organ failures. Biomarkers to reliably predict the patient’s clinical cause of the virus infection, ideally, to be applied in point of care testing or through routine diagnostic approaches, are highly needed. We aimed to probe if routinely assessed clinical lab values can predict the severity of the COVID-19 course. Therefore, we have retrospectively analyzed on admission laboratory findings in 224 consecutive patients from four hospitals and show that systemic immune inflammation index (SII) is a potent marker for predicting the requirement for invasive ventilator support and for worse clinical outcome of the infected patient. Patients’ survival and severity of SARS-CoV-2 infection could reliably be predicted at admission by calculating the systemic inflammatory index of individual blood values. We advocate this approach to be a feasible and easy-to-implement assay that may be particularly useful to improve patient management during high influx crisis. We believe with this work to contribute to improving infrastructure availability and case management associated with COVID-19 pandemic hurdles.

## 1. Introduction

The number of SARS-CoV-2-infected patients is increasing dramatically and has now reached over 75 million confirmed infected individuals with over 1.6 million deaths worldwide (John Hopkins Corona Resource Center https://coronavirus.jhu.edu/about). The disease was first reported in December 2019 in the city of Wuhan, China with a cluster of pneumonia of unknown origin. In January 2020, the disease spread to Japan and over February/March 2020 to Europe, before spreading globally in spring 2020. Intensive epidemiological and biological research led to the advancement of our understanding of the virus biology with various clinical trials for therapeutic vaccines underway [1,2] (https://clinicaltrials.gov/ct2/who_table). SARS-CoV-2 infection is primarily respiratory and can range from common cold with mild symptoms to severe acute respiratory syndrome (SARS) with respiratory failure requiring invasive respiratory support [3,4] eventually leading to death in the worst case. Regular hygiene, wearing a face mask and social distancing measures are the only available, but not targeted, strategies to minimize the spread of the disease. Limited effectivity of the aforementioned measures, compounded with occasional seasonal and regional peaks in the number of COVID-19 cases also leads to a significant, but unsteady demand for ventilation/intensive care beds. Under such circumstances, even the most developed Western world health systems are challenged, leading to disastrous scenarios with high mortality related to SARS-CoV-2 infection. Reliable biomarkers predicting a mild or dismissal course of a patient are of high clinical and economical importance.

Some patients suffering from COVID-19 have been found to present exaggerated systemic inflammatory response [5] with numerous molecular routes how the virus impedes the host immune answer been published [6,7]. However, there is no consensus regarding the utility of a quantitative analysis of the systemic immune cell composition of COVID-19 patients. The systemic immune inflammation index (SII, (#neutrophils × #platelets)/#lymphocytes) is a predictive parameter for the diagnosis of cancer [8,9,10]. We exploited this easy-to-perform and rapid-to-calculate parameter on patient admission in the context of SARS-CoV-2 infection and investigated whether SII can help in predicting the clinical progression of the patient. Our results are statistically highly significant and we believe this data can be useful for the global fight of the COVID-19 pandemic, particularly in the commencement of the current phase of resurge of infected cases risking a high load of patients in hospitals.

## 2. Results

### 2.1. Patients Characteristics

Only cases with a confirmed viral infection, as defined by a positive PCR test result on a nasal-swab sample, were included. In many cases, diagnostics was followed with computer tomography. Of the 224 patients, 77, 25, 62 and 60 came from the hospital nr. 1, 2, 3, and 4, respectively. The patients’ mean age was 62 ± 17.131 (58%) were male. Following comorbidities were recorded: 65 (29%) of the patients suffered from diabetes, 80 (36%) from hypertension, 32 (14%) from heart failure, 16 (7%, one missing value) from COPD, 18 (8%) from a stroke. Regarding medication, 53 (24%) required acetylsalicylic acid, 55 (25%) beta-blockers, 70 (31%) ACE inhibitors, and 60 (27%) anti-diabetic medication. 91 patients (41%, one missing) required intubation during hospitalization. During their hospital stay, 13 (11%, 101 missing) suffered liver failure, 59 (26%) kidney failure, 15 (7%) heart failure, and 3 (1%) stroke. 68 of the patients (30%) did not survive.

Among the surviving patients, 95 (61%) were male, while among the deceased, only 34 (50%) were male. The surviving patients were generally younger (58 ± 17 years) than the deceased (72 ± 13). The lab values for all patients are listed in Table 1.

All ethical and legal required formalities according to Iranian national guidelines to conduct this retrospective study have been granted for this work, and are in concordance to international standards such as the declaration of Helsinki.

### 2.2. Predicting Intubation and Outcome

**Figure 1:**
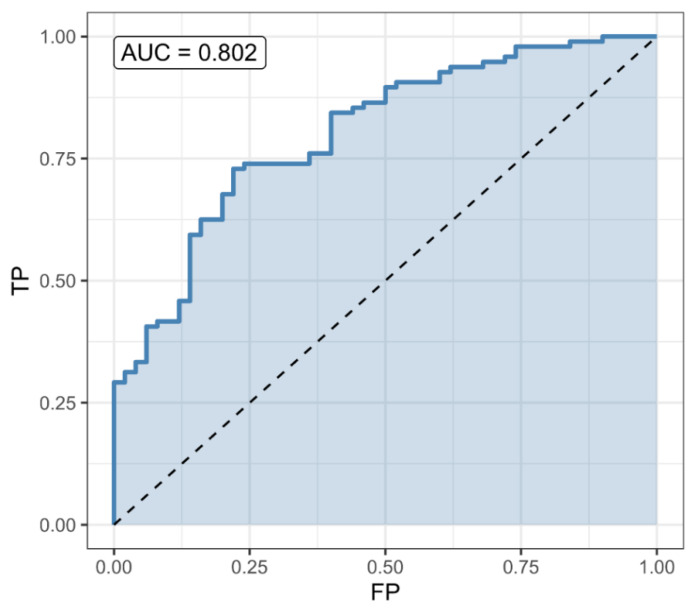
A predictive model for survival a: receiver operating characteristic (ROC) curve for the survival predictor, based on age, systemic immune inflammation index (SII), antidiabetics and beta-blockers. The predictor is significantly better than the non-informative (null) predictor (*p* = 0.0026). A classifier based on the Pr(survival) = 0.5 threshold had a sensitivity of 0.875, specificity of 0.571, and accuracy of 0.772 (CI = (0.695, 1)). Its accuracy was significantly better (*p* = 0.0026) than the noninformative model (accuracy = 0.662).

**Figure 2:**
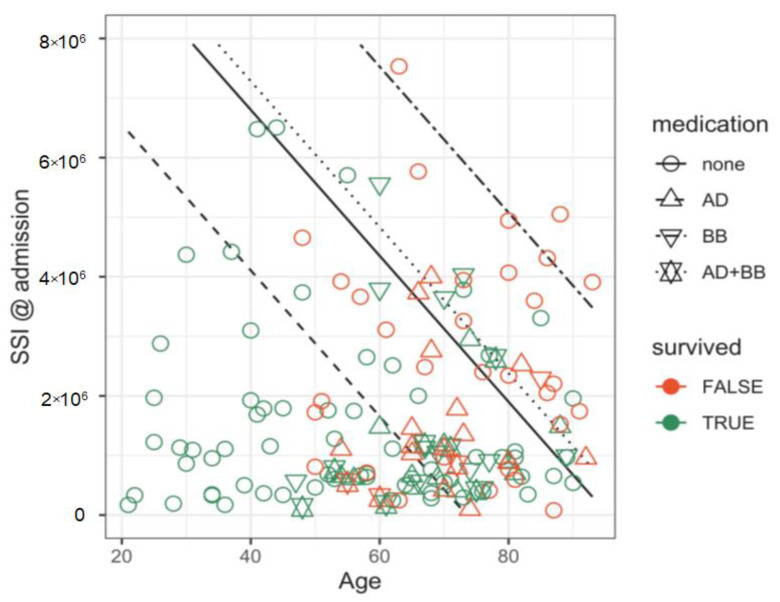
A predictive model for survival b: patient data and predicted boundaries for 50% survival probabilities. Younger patients and those with a lower SII at admission (lower left area of the figure) have much better survival chances than older patients and patients with a high SII (upper right area). The four black lines denote 50% survival boundaries for four possible combinations of medications taken by the patients: none, antidiabetics (AD), beta-blockers (BB) and both antidiabetics and beta-blockers (AD + BB).

A classifier based on the Pr(survival) = 0.5 threshold had a sensitivity of 0.875, specificity of 0.551, and accuracy of 0.766 (CI = (0.688, 1)). Its accuracy was significantly better (*p* = 0.0045) than the noninformative model (accuracy = 0.662) (Figure 1). Patients’ survival could be reliably predicted at admission. Step-wise variable selection led to a model with age (OR = 0.95, CI = (0.93, 0.98), *p* = 0.00011), the logarithm of SII (OR = 0.18, CI = (0.05, 0.66), *p* = 0.01), antidiabetics (OR = 0.36, CI = (0.13, 0.99), *p* = 0.048) and beta blockers (OR = 3.82, CI = (0.89, 16.38), *p* = 0.071) as predictors for survival. McFadden’s pseudo-R^2^ was 0.205 (Figure 2).

The data projected on the age/log-SII plane with the corresponding 50% survival boundaries for all combinations of significant medication are shown in supplement files (Supplementary Materials Figures S1 and S2).

**Figure 3:**
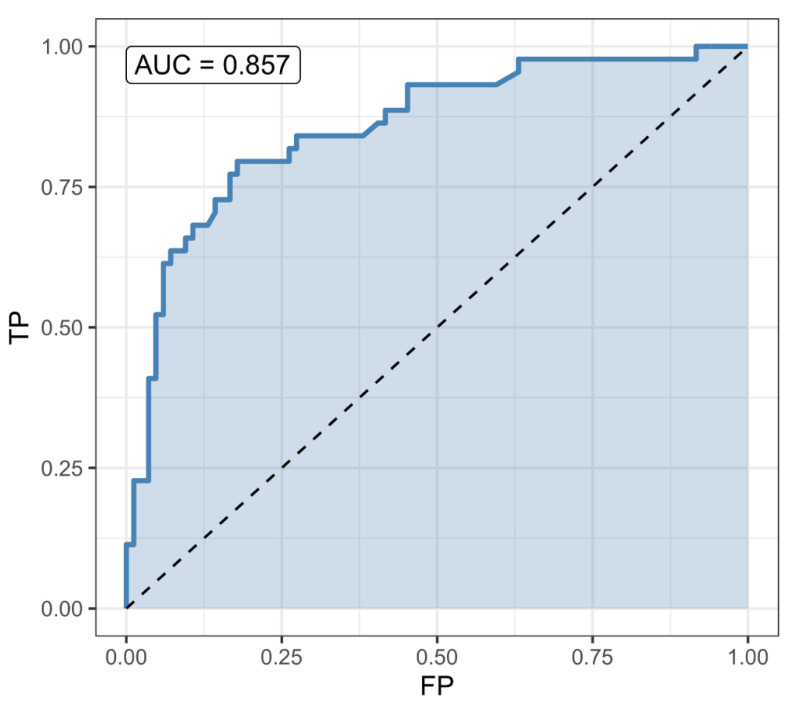
Predictions for complications: ROC curve for the kidney failure predictor, based on monocytes, creatinine, and alkaline phosphatase at admission. The predictor is better than the non-informative (null) predictor, but not significantly (*p* = 0.13). A classifier based on the Pr(kidney failure) = 0.5 threshold had a sensitivity of 0.455, specificity of 0.987, and accuracy of 0.919 (CI = (0.839, 0.967)). However, since few patients developed kidney failure (11 out of 86 = 12.8%), its accuracy was not significantly better (*p* = 0.13) than the noninformative model’s (accuracy = 0.872).

Among the complications, only kidney failure could be reliably predicted using available data. Forward step-wise selection led to a model using creatinine (OR = 1.002, CI = (1, 1.004), *p* = 0.079), monocytes (OR = 4.62, CI = (1.58, 13.47), *p* = 0.0051), and alkaline phosphatase (OR = 1.012, CI = (1, 1.023), *p* = 0.041) (all at admission) as predictors. McFadden’s pseudo-R^2^ was 0.355. A classifier based on the Pr(kidney failure) = 0.4 threshold had a sensitivity of 0.455, specificity of 0.947, and accuracy of 0.884 (CI = (0.797, 0.943)). However, since few patients developed kidney failure (11 out of 86 = 12.8%), its accuracy was not significantly better (*p* = 0.45) than the noninformative model’s (accuracy = 0.872) (Figure 3).

**Figure 4:**
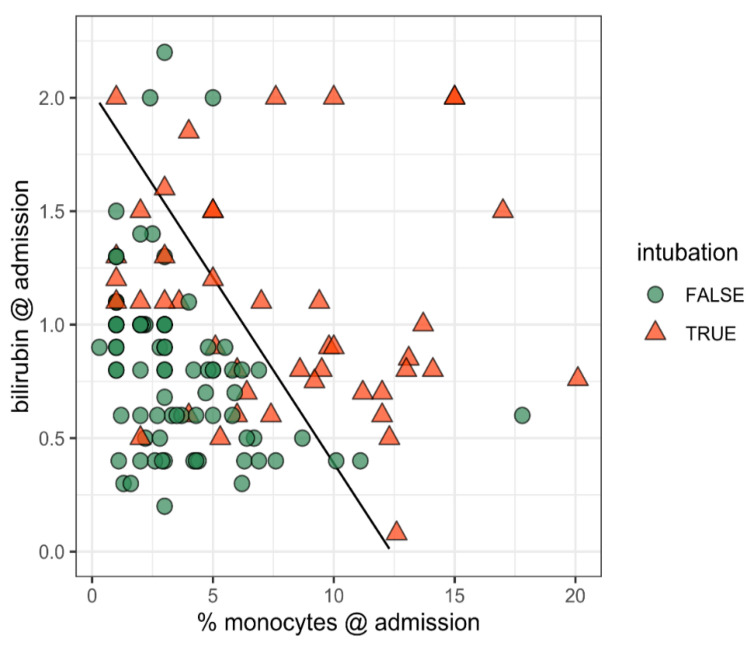
The predicting intubation requirement a: patient data and the predicted boundary (black line) for 50% intubation probabilities. Patients with a lower percentage of monocytes and lower bilirubin levels at admission (lower left area of the figure) are much less likely to require intubation.

**Figure 5:**
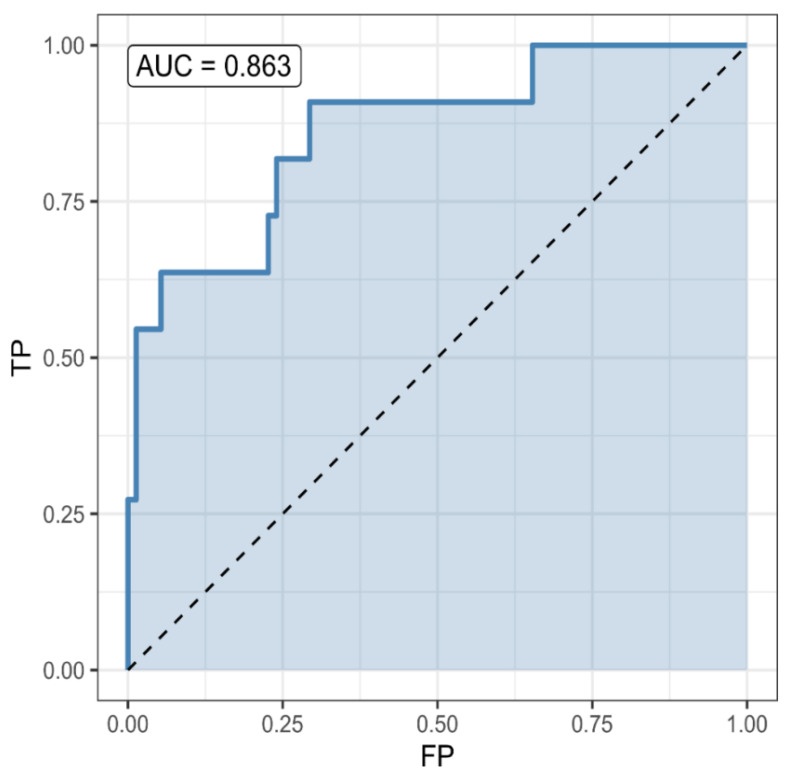
The predicting intubation requirement b: ROC curve for the intubation predictor, based on % monocytes and bilirubin at admission. The predictor is significantly better than the non-informative (null) predictor (*p* = 3.1 × 10^−5^). A classifier based on the Pr(intubation) = 0.5 threshold had a sensitivity of 0.614, specificity of 0.929, and accuracy of 0.82 (CI = (0.743, 0.883)). Its accuracy was significantly better (*p* = 3.1× 10^−5^) than the noninformative model’s (accuracy = 0.656).

Whether a patient would require intubation could also be reliably predicted at admission. The data projected on the %-monocytes/bilirubin plane with the 50% boundary for intubation are shown in Figure 4. Step-wise variable selection led to a model with percentage of monocytes (OR = 1.48, CI = (1.27, 1.73), *p* = 8.9 × 10^−7^), and bilirubin (OR = 11.06, CI = (3.11, 39.35), *p* = 0.00021) as predictors. McFadden’s pseudo-R^2^ was 0.308 (Figure 5).

## 3. Discussion

COVID-19 pandemic is a global health problem affecting all societies globally. Improved management of the COVID-19 disease, both in the therapeutic and predictive diagnostic sector, is needed to fight this urgent problem. Our work is in line with the global efforts to develop better diagnostics for the disease ranging from the development of infection detection assays [11,12] to disease progression surveillance assays [13] as well as predictive diagnostics [14,15]. Our work extends the lists of inflammation-associated proposals for predictive biomarkers for the course of SARS-CoV-2 infections. As such, the respiratory inflammation index [16,17], cytokine signature [18,19,20], hyper inflammation signature [21,22], lymphocyte subsets [23,24,25], diet-instructed immune index [26], amongst others (the authors acknowledge the rapidly evolving literature on this topic, making it impossible to comprehensively cite all relevant work), have been proposed to predict severity of the disease course. As a remark, our results are in line with very recently published work identifying the value of SSI to predict the risk of in-hospital mortality of COVID-19 patients [27]. Notwithstanding this growing list of prediction assays, we believe our work is important for the world and significantly distinct from previous work for three main reasons: First, given the striking significant clinical prognostic value in COVID-19 patient population from different treatment centers, we strongly believe in the robustness of our proposed application. Second, since SII can be simply calculated from blood sample data routinely acquired around the world, our application has great potential to be rapidly implemented and thereby rapidly disseminated worldwide. This is not only in relation to the aforementioned inflammation markers, but especially in comparison to attempts to predict COVID-19 severity based on imaging diagnostic, which require special infrastructure and trained staff [28]. The latter fact makes our approach distinct and more suitable for dissemination as compared to other prediction-oriented assays. Lastly, in comparison to [27], our work extends the relevance of SSI at hospital admission to predicting the course of the disease to non-lethal outcome measurements. Our work confirms the results of [27], presenting data of a similar-sized patient cohort, but also extends the relevance of SSI as a powerful predictive diagnostics in COVID-19 patients to Middle Eastern ethnicity. Confirmatory clinical studies, ideally multicentre studies in different ethnic backgrounds, represent a cornerstone of establishing new clinical guidelines [29], and our work adds the value of using SSI at hospital admission for managing COVID-19 patients. Being based on simple quantification of cell composition, our blood-based diagnostics is suitable for application even in centers with minimal resource availability, such as rural areas or without sophisticated lab infrastructure. The authors acknowledge that the presented data does not include the analysis of widely used, infections-indicating blood parameters, such as C-reactive protein or others. The inclusion of those parameters in addition to the SSI may increase the specificity or predictive power of our assay, but this is not the scope of this study. In addition to its clinical relevance, we furthermore hypothesize that SSI–based stratification of COVID-19 patients may be useful in designing clinical trials regarding the disease. Depending on the trial design, patients can be selected or excluded according to the predicted severity of the course. We appreciate the great potential of artificial intelligence-guided prediction of disease progression based on various other routinely available clinical data [30]. Thus, implementation of SSI in such multi-parameter computational-based predictions may further improve the power of such machine—learning-based approaches for COVID-19 patient management. The authors furthermore acknowledge the great potential of modern biosensors for improving diagnostics of SARS-CoV-2 infection including their use in predictive medicine [31]. Here, CRISPR/Cas-based diagnostics seem most potent for rapid application due to their target amplification-free set-up and high accuracy [32] making them ideal for POCT. However, as of now, no standardized CRISPR/Cas diagnostics are available and the field still lags behind routine diagnostics (such as SSI) in terms of long-term clinical experience. The focus of our work lays in providing diagnostics that can predict the severity course of the disease at admission. Including the duration of stay, or other later-time point related data, in the statistical model, would not be relevant for acute management of new admissions. Our study is limited by the total number and only a single ethnicity of the included patients. We propose the independent validation of our findings in other parts of the world, ideally in prospective clinical settings. This will also tackle the problem of using a subpopulation of the overall patient cohort of our study for validation purposes, which was due to the limited number of available independent patient cohorts.

## 4. Material and Methods

Clinical and on-admission laboratory data were collected retrospectively from four hospitals treating COVID-19 patients. 224 patients with complete records were identified. Patients’ characteristics, clinical and laboratory data were analyzed using R, version 3.6.1 “Action of the Toes”. Missing values were removed. For descriptive statistics, the percentages were calculated using only available data. Odds ratios between comorbidities and medications (uncorrected) were computed from contingency tables and tested using Fisher’s exact test. Multivariable logistic regression was used for modeling survival probability, probability of complications, and intubation probability. Predictor variables were chosen by step-wise forward selection, based on Akaike’s Information Criterion (AIC). After having selected the predictor variables, the models were retrained and validated using leave-one-out validation. The significance level of 0.05 was used.

According to national rules, retrospective analysis of patient samples that are routinely assessed and acquired (not requiring extra effort for acquisition) do not require explicit ethical approval. For details about national biomedical research guidelines, see [33].

## 5. Conclusions

Systemic immune inflammation index is an easy-to-quantify parameter with high sensitivity and specificity to predict the clinical course of SARS CoV-2 infected patients. SII might be a valuable tool to improve the management of COVID-19.

## Figures and Tables

**Table 1:** General lab values of patient cohort.

	At Admission		At Intubation	
Lab	Mean	SD	Mean	SD
Platlets	187,000	89,633	178,316	97,356
WBC	9076	5639	9968	7134
Neutrophils (%)	75.9	12.7	83.2	8.4
Lymphocytes (%)	18.6	11.5	15.2	27.9
Monocytes (%)	4.33	3.91	7.5	8.3
Neutrophils (/µL)	7941	6228	10,894	6003
Lymphocytes (/µL)	1165	629	1093	673
Monocytes (/µL)	503	431	1626	2377
Creatining	1.76	1.63	3.82	2.34
SGOT	46.1	39.5	65.8	30.8
SGPT	35.2	35.3		
Alkaline Phospathase	212.8	232.5	278	122
Bilirubin	0.90	0.43	2.21	1.00
AST			56.9	29.1
SII	1,769,543	1,610,378	2,087,603	1,592,935

## Data Availability

The data presented in this study are available on request from the corresponding author. The data are not publicly available due to patient privacy and patint ethical consideration.

## Supplementary Materials

The following are available online at https://www.mdpi.com/2076-0817/10/1/58/s1, Figure S1: SII-Survival relationship of individuals included in this study. Figure S2: Age-Survival relationship of individuals included in this study.

## References

[1] Asghari A., Naseri M., Safari H., Saboory E., Parsamanesh N. (2020). The Novel Insight of SARS-CoV-2 Molecular Biology and Pathogenesis and Therapeutic Options. DNA Cell Biol..

[2] Karlsen A.P.H., Wiberg S., Laigaard J., Pedersen C., Rokamp K.Z., Mathiesen O. (2020). A Systematic Review of Trial Registry Entries for Randomized Clinical Trials Investigating COVID-19 Medical Prevention and Treatment. PLoS ONE.

[3] Wang D., Hu B., Hu C., Zhu F., Liu X., Zhang J., Wang B., Xiang H., Cheng Z., Xiong Y. (2020). Clinical Characteristics of 138 Hospitalized Patients With 2019 Novel Coronavirus–Infected Pneumonia in Wuhan, China. JAMA.

[4] Guan W., Ni Z., Hu Y., Liang W., Ou C., He J., Liu L., Shan H., Lei C., Hui D.S.C. (2020). Clinical Characteristics of Coronavirus Disease 2019 in China. N. Engl. J. Med..

[5] Lucas C., Wong P., Klein J., Castro T.B.R., Silva J., Sundaram M., Ellingson M.K., Mao T., Oh J.E., Israelow B. (2020). Longitudinal Analyses Reveal Immunological Misfiring in Severe COVID-19. Nature.

[6] Chen Z., John Wherry E. (2020). T Cell Responses in Patients with COVID-19. Nat. Rev. Immunol..

[7] Chowdhury M.A., Hossain N., Kashem M.A., Shahid M.A., Alam A. (2020). Immune Response in COVID-19: A Review. J. Infect. Public Health.

[8] Zhang Y., Sun Y., Zhang Q. (2020). Prognostic Value of the Systemic Immune-Inflammation Index in Patients with Breast Cancer: A Meta-Analysis. Cancer Cell Int..

[9] Chen J.-H., Zhai E.-T., Yuan Y.-J., Wu K.-M., Xu J.-B., Peng J.-J., Chen C.-Q., He Y.-L., Cai S.-R. (2017). Systemic Immune-Inflammation Index for Predicting Prognosis of Colorectal Cancer. World J. Gastroenterol..

[10] Topkan E., Besen A.A., Ozdemir Y., Kucuk A., Mertsoylu H., Pehlivan B., Selek U. (2020). Prognostic Value of Pretreatment Systemic Immune-Inflammation Index in Glioblastoma Multiforme Patients Undergoing Postneurosurgical Radiotherapy Plus Concurrent and Adjuvant Temozolomide. Mediat. Inflamm.

[11] De Moura D.T.H., McCarty T.R., Ribeiro I.B., Funari M.P., de Oliveira P.V.A.G., de Miranda Neto A.A., do Monte Júnior E.S., Tustumi F., Bernardo W.M., de Moura E.G.H. (2020). Diagnostic Characteristics of Serological-Based COVID-19 Testing: A Systematic Review and Meta-Analysis. Clinics.

[12] Ferrari D., Motta A., Strollo M., Banfi G., Locatelli M. (2020). Routine Blood Tests as a Potential Diagnostic Tool for COVID-19. Clin. Chem. Lab. Med..

[13] Tong X., Ning M., Huang R., Jia B., Yan X., Xiong Y., Wu W., Liu J., Chen Y., Wu C. (2020). Surveillance of SARS-CoV-2 Infection among Frontline Health Care Workers in Wuhan during COVID-19 Outbreak. Immun. Inflamm. Dis..

[14] Harahwa T.A., Yau T.H.L., Lim-Cooke M.-S., Al-Haddi S., Zeinah M., Harky A. (2020). The Optimal Diagnostic Methods for COVID-19. Diagnosis.

[15] Grau C.M., Bofill C.B., Picó-Plana E., Comí G.R., Terrón-Puig M., Paz N.B., Mateu M.S., Fornés C.G. (2020). Use of Predictive Tools in the Management of COVID-19 Patients: A Key Role of Clinical Laboratories. Adv. Lab. Med. Av. Med. Lab..

[16] Mohamed-Hussein A., Galal I., Mohamed M.M.A.R., Elaal H.A., Aly K.M. (2020). Is There a Correlation between Pulmonary Inflammation Index with COVID-19 Disease Severity and Outcome?. medRxiv.

[17] Wan S., Yi Q., Fan S., Lv J., Zhang X., Guo L., Lang C., Xiao Q., Xiao K., Yi Z. (2020). Relationships among Lymphocyte Subsets, Cytokines, and the Pulmonary Inflammation Index in Coronavirus (COVID-19) Infected Patients. Br. J. Haematol..

[18] Del Valle D.M., Kim-Schulze S., Huang H.-H., Beckmann N.D., Nirenberg S., Wang B., Lavin Y., Swartz T.H., Madduri D., Stock A. (2020). An Inflammatory Cytokine Signature Predicts COVID-19 Severity and Survival. Nat. Med..

[19] Angioni R., Sánchez-Rodríguez R., Munari F., Bertoldi N., Arcidiacono D., Cavinato S., Marturano D., Zaramella A., Realdon S., Cattelan A. (2020). Age-Severity Matched Cytokine Profiling Reveals Specific Signatures in Covid-19 Patients. Cell Death Dis..

[20] Huang C., Wang Y., Li X., Ren L., Zhao J., Hu Y., Zhang L., Fan G., Xu J., Gu X. (2020). Clinical Features of Patients Infected with 2019 Novel Coronavirus in Wuhan, China. Lancet.

[21] Manson J.J., Crooks C., Naja M., Ledlie A., Goulden B., Liddle T., Khan E., Mehta P., Martin-Gutierrez L., Waddington K.E. (2020). COVID-19-Associated Hyperinflammation and Escalation of Patient Care: A Retrospective Longitudinal Cohort Study. Lancet Rheumatol..

[22] Gustine J.N., Jones D. (2021). Immunopathology of Hyperinflammation in COVID-19. Am. J. Pathol..

[23] Jiang M., Guo Y., Luo Q., Huang Z., Zhao R., Liu S., Le A., Li J., Wan L. (2020). T-Cell Subset Counts in Peripheral Blood Can Be Used as Discriminatory Biomarkers for Diagnosis and Severity Prediction of Coronavirus Disease 2019. J. Infect. Dis..

[24] Zhang W., Li L., Liu J., Chen L., Zhou F., Jin T., Jiang L., Li X., Yang M., Wang H. (2020). The Characteristics and Predictive Role of Lymphocyte Subsets in COVID-19 Patients. Int. J. Infect. Dis..

[25] Deng Z., Zhang M., Zhu T., Zhili N., Liu Z., Xiang R., Zhang W., Xu Y. (2020). Dynamic Changes in Peripheral Blood Lymphocyte Subsets in Adult Patients with COVID-19. Int. J. Infect. Dis..

[26] Calder P.C. (2020). Nutrition, Immunity and COVID-19. BMJ Nutr. Prev. Health.

[27] Fois A.G., Paliogiannis P., Scano V., Cau S., Babudieri S., Perra R., Ruzzittu G., Zinellu E., Pirina P., Carru C. (2020). The Systemic Inflammation Index on Admission Predicts In-Hospital Mortality in COVID-19 Patients. Molecules.

[28] Feng Z., Yu Q., Yao S., Luo L., Zhou W., Mao X., Li J., Duan J., Yan Z., Yang M. (2020). Early Prediction of Disease Progression in COVID-19 Pneumonia Patients with Chest CT and Clinical Characteristics. Nat. Commun..

[29] Stallard N., Todd S., Parashar D., Kimani P.K., Renfro L.A. (2019). On the Need to Adjust for Multiplicity in Confirmatory Clinical Trials with Master Protocols. Ann. Oncol..

[30] Yan L., Zhang H.-T., Goncalves J., Xiao Y., Wang M., Guo Y., Sun C., Tang X., Jin L., Zhang M. (2020). A Machine Learning-Based Model for Survival Prediction in Patients with Severe COVID-19 Infection. medRxiv.

[31] Morales-Narváez E., Dincer C. (2020). The Impact of Biosensing in a Pandemic Outbreak: COVID-19. Biosens. Bioelectron..

[32] Broughton J.P., Deng X., Yu G., Fasching C.L., Servellita V., Singh J., Miao X., Streithorst J.A., Granados A., Sotomayor-Gonzalez A. (2020). CRISPR–Cas12-Based Detection of SARS-CoV-2. Nat. Biotechnol..

[33] Mardani A., Nakhoda M., Noruzi A., Shamsi Gooshki E. (2019). Ethical Considerations in the Biomedical Research: Analysis of National Biomedical Research Ethics Guidelines in Iran. J. Med. Ethics Hist. Med..

