# Supplementary material for: Systemic Inflammatory Index Is a Novel Predictor of Intubation Requirement and Mortality after SARS-CoV-2 Infection
Source: Pathogens. 2021 Jan 11;10(1):58. doi: 10.3390/pathogens10010058 (PMC7827801; doi:10.3390/pathogens10010058)

Supplementary Figure S1: Percentage survival of patients in association of their SSI at time of hospital admission.

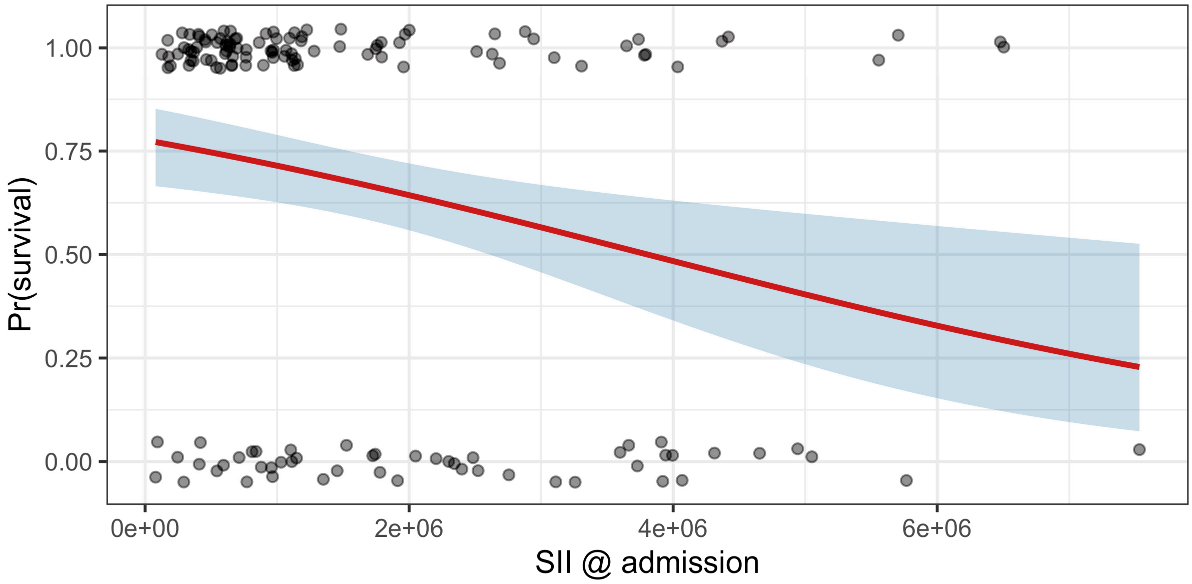

Supplementary Figure S2: Percentage survival of patients in association of their age at time of hospital admission. Stratification according to gender of patients (f- female, m- male).

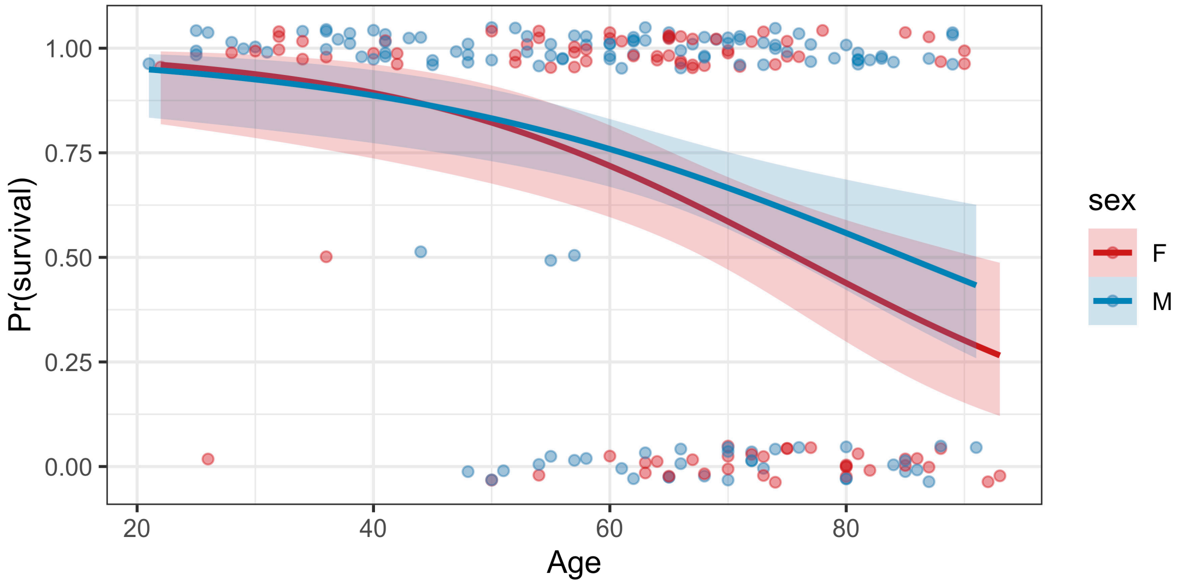

Supplement: Supplementary file 1 [file pathogens-10-00058-s001.pdf]
